# Impact of the COVID-19 Outbreak on the Treatment of Myocardial Infarction Patients

**DOI:** 10.1007/s11936-023-00988-3

**Published:** 2023-06-09

**Authors:** Maik J. Grundeken, Bimmer E. P. M. Claessen

**Affiliations:** Department of Cardiology, Heart Center, Amsterdam UMC, University of Amsterdam, Amsterdam Cardiovascular Sciences, 1105 AZ Amsterdam, Netherlands

**Keywords:** SARS-CoV-2, COVID-19, Pandemic, ACS, NSTEMI, STEMI

## Abstract

**Purpose of review:**

The COVID-19 pandemic has led to an overburdened healthcare system. While an increased rate of ACS is expected due to the pro-thrombotic state of COVID patients, observed ACS incidence and admission rates were paradoxically decreased during the (first wave of the) pandemic. In this narrative review, we will discuss potential reasons for this decrease in ACS incidence. Furthermore, we will discuss ACS management during the COVID-19 pandemic, and we will discuss outcomes in ACS.

**Recent findings:**

A reluctance to seek medical contact (in order not to further overburden the health system or due to fear of being infected with COVID-19 while in hospital) and unavailability of medical services seem to be important factors. This may have led to an increased symptom onset to first medical contact time and an increased rate of out-of-hospital cardiac arrests. A trend towards less invasive management was observed (less invasive coronary angiography in NSTEMI patients and more “fibrinolysis-first” in STEMI patients), although a large variation was observed with some centers having a relative increase in early invasive management. Patients with ACS and concomitant COVID-19 infection have worse outcomes compared to ACS patients without COVID-19 infection. All of the above led to worse clinical outcomes in patients presenting with ACS during the COVID-19 pandemic. Interestingly, staffing and hospital bed shortages led to experimentation with very early discharge (24 h after primary PCI) in low-risk STEMI patients which had a very good prognosis and resulted in significant shorter hospital duration.

**Summary:**

During the COVID-19 pandemic, ACS incidence and admission rates were decreased, symptom onset to first medical contact time prolonged, and out-of-hospital rates increased. A trend towards less invasive management was observed. Patients presenting with ACS during the COVID-19 pandemic had a worse outcome. On the other hand, experimental very early discharge in low-risk patients may relieve the healthcare system. Such initiatives, and strategies to lower the reluctance of patients with ACS symptoms to seek medical help, are vital to improve prognosis in ACS patients in future pandemics.

## Introduction

In late 2019, cases of pneumonia with an unknown etiology in Wuhan were reported by the Chinese Health Authority. A novel virus was identified, initially called 2019-nCOV (novel coronavirus 2019). After further research revealed its association with the coronavirus (CoV) which caused an outbreak of severe acute respiratory syndrome (SARS) in 2002, the novel virus was renamed SARS-CoV-2 [1]. The disease caused by this new virus was called coronavirus disease of 2019 (COVID-19) and spread rapidly causing a large burden of morbidity and mortality worldwide. The World Health Organization (WHO) declared the worldwide spread as a pandemic on March 11^th^ [2].

Acute coronary syndrome (ACS) encompasses unstable angina, non-ST-segment elevation myocardial infarction (NSTEMI), and ST segment elevation myocardial infarction (STEMI). Its clinical presentation is broad and ranges from cardiac arrest, cardiogenic shock, ventricular arrhythmias, or painfree at presentation [3]. Its leading symptom is acute chest discomfort (e.g., pain, pressure, tightness, or burning), and it is caused by myocardial ischemia, and ultimately necrosis, due to coronary stenosis with or without plaque rupture and local thrombosis [3]. Its acute treatment includes antithrombotic medication and coronary revascularization and for the longer term preventive measures including lifestyle changes and secondary preventive medication [3].

In this narrative review, we will discuss the impact of the COVID-19 pandemic on ACS, including the impact on incidence, diagnosis, treatment, and prognosis.

## Influence of the COVID-19 Pandemic on ACS Incidence

### Hypercoagulopathy in COVID-19

Early in the pandemic, venous and arterial thrombotic complications were noted in patients admitted with COVID-19 including deep venous thrombosis, pulmonary embolus, acute limb ischemia, acute cerebrovascular accident, aortic thrombosis, mesenteric ischemia, disseminated intravascular coagulation, and acute coronary syndrome [4•]. The pathophysiology is partly understood. SARS-CoV2 binds to angiotensin-converting enzyme-2 (ACE-2). ACE-2 acts as a counter-regulatory enzyme-converting angiotensin I to angiotensin II and is present throughout the human body including endothelial cells in the small and large arteries and veins and alveolar epithelial cells in the lungs and in nasal, oral, and nasopharynx mucosa cells [5]. By binding to ACE-2, SARS-CoV2 inhibits its function, preventing angiotensin I to be broken down by ACE-2, promoting a pro-inflammatory state, as well as vasoconstriction, sodium retention and fibrosis [6]. The pro-inflammatory milieu is evidenced by high levels of cytokines IL-2, IL-7, IL-10, and IgG-induced protein 10, granulocyte-stimulating factor, macrophage inflammatory protein 1-alpha, monocyte chemoattractant protein-1, and tumor-necrosis factor alpha [7]. This inflammation may result in an hypercoagulable state via endothelial cells dysfunction, activating platelets and tissue factor (TF), triggering the coagulation by binding of TF to factor VIIa [8, 9], leading to venous and arterial thrombosis.

### COVID-19-induced ACS

Two types of myocardial infarction may occur in patients with COVID-19: type 1 myocardial infarction (caused by a critical coronary stenosis with or without plaque rupture and local thrombosis) and type 2 myocardial infarction (caused by “demand ischemia” due to hypoxemia, hypotension, anemia, and with or without underlying stable coronary artery disease) [3].

As outlined above, COVID-19 infection resulting in a pro-inflammatory state may lead to a higher propensity of plaque rupture [10] and thrombus formation [11] leading to type I acute myocardial infarction. Indeed, concomitant occurrence of ACS in patients with COVID-19 has been reported [12, 13•, 14–17]. These data suggest that COVID-19 patients presenting with ACS have a high thrombus burden including multivessel thrombus and stent thrombosis [13•] and, when compared to historical controls without COVID-19, have worse outcome with a higher risk for cardiogenic shock and increased risk of mortality [16]. These differences might be explained by differences in coagulability [12] but also increased symptom-to-admission times [16]. However, these data need to be interpreted with caution since study size is relatively small and only few studies have collected data prospectively.

### Decreased ACS Admission Rates

Despite the above outlined correlation between COVID-19 infection and occurrence of venous and arterial thrombosis including ACS, a surprisingly substantial reduction in ACS admission rates was observed since the COVID-19 pandemic was declared. A reduction in STEMI presentations of 2.4% to as high as 48.9% was observed [18–20]. A systematic review including 11 studies with STEMI patients showed an overall reduction of 24% [21]. In NSTEMI, this was 40 to 50% during the first wave [19, 20, 22–25]. A meta-analysis including 9 studies on patients admitted with NSTEMI during the first wave showed an overall reduction of 31% [21]. Multiple studies have revealed an upward trend towards normal at the end of this first wave [19, 26].

Although not completely understood, multiple explanations were proposed, which can be divided in two subgroups: (1) a true lower overall incidence of ACS and (2) ACS underdiagnosing due to patient behavior and/or an overburdened healthcare system.

*A true lower overall incidence* might be explained by several reasons. Early in the pandemic, during lockdowns, patients might have been exposed to less physical and emotional stress, triggering less ACS. Sport facilities were closed, and in some countries, there was an evening curfew, limiting the possibilities to perform (vigorous) exercise. Other well-known potential triggers such as exposure to polluted air and other respiratory disease such as the flu were also reduced [27–29]. Furthermore, they might have improved their health behavior practice to help alleviate healthcare burden [30].

*ACS underdiagnosing due to patients’ behavior and an overburdened healthcare system* may be another explanation. Patients were reluctant to burden the healthcare system and seek medical help. Furthermore, they may have been reluctant to avoid contact with COVID-19 patients at the emergency departments. Indeed, symptom onset to first medical contact times was increased [21]. Furthermore, out-of-hospital cardiac arrests were increased, which could not all be attributed to COVID-19 infections, suggesting primary cardiac arrests in patients with ACS not seeking medical help [31•].

## Symptom Onset-to-Treatment Time in STEMI Patients

Symptom onset to treatment time in ACS during the pandemic may have been influenced by the above-mentioned overburdened healthcare system and possible reluctance to seek medical attention. Studies have indeed shown increased symptom onset to first medical contact time [32], symptom onset to door time [33], and symptom onset to balloon time [34]. A meta-analysis of 28 studies published in 2022 showed an average increase in symptom onset to first medical contact time of 91 min during the first wave in 2020 when compared to the same period in 2019 [21].

In-hospital delay appears to have been only limited as other studies have shown door-to-balloon time was not increased [35, 36]. The aforementioned meta-analysis including 24 studies which reported door-to-balloon times showed a statistical significant but in absolute terms only modest increase in door-to-balloon time of 5 min [21].

## Treatment Strategies During the Pandemic

In parallel to the previously described decline in ACS presentation and admission rates, a drop in PCI rates was observed. In a large study performed in the UK, PCI in STEMI patients decreased with 18% and PCI in NSTEMI patients with 37% [20]. However, from the patients admitted for ACS, the proportion of patients undergoing PCI was slightly increased during the first wave of the pandemic [20]. A Chinese study on the other hand showed a lower percentage of invasive strategy in patients presenting with NSTEMI early in the pandemic period as compared with NSTEMI patients presenting in the same period one year earlier [37]. It may therefore be hypothesized that the impact of the pandemic on treatment strategies was not uniform across all geographies but rather dependent on several factors including local healthcare protocols, geographical factors (rural vs. urban agglomerations), and intensity of the pandemic expressed in the number of infected and hospitalized.

A “fibrinolysis-first” treatment strategy (instead of primary PCI) was proposed as an alternative strategy for acute revascularization in patients presenting with STEMI to alleviate the overburdened healthcare system during the COVID-19 pandemic [38, 39]. This strategy however led to a lower rate of timely reperfusion with an increased rate of recurrent ischemia and a higher rate of cardiogenic shock, and more patients developed heart failure in a Chinese hospital in Beijng [38]. Another study performed in a hospital in Tianjin, China, compared treatment strategies early in the COVID-19 pandemic period (January to February 2020) with the same period one year earlier [37]. A primary PCI strategy was performed more often in the pre-COVID-19 pandemic period, while thrombolytic therapy was performed more often in the early pandemic period [37]. This did not result in differences in 30-day all-cause mortality or major cardiac adverse event rates in STEMI patients presenting during the different periods [37].

## Length of Hospital Admission

During the pandemic, especially during the first wave, the length of hospital admissions was reduced for both NSTEMI and STEMI, as shown by several studies. A large study in the UK showed a reduction in median admission time from 5 days (interquartile range 3–11) to 3 days (IQR 2–6) for NSTEMI and 3 days (IQR 2–6) to 2 days (IQR 2–4) in STEMI patients [20]. An analysis from the Mass General Brigham health system revealed that length of stay for patients admitted for ACS was significantly shorter during the first pandemic wave (March 2020) compared with the same period 1 year earlier (median of 4.8 days [IQR 2.4–8.3 days] versus 6.0 days [IQR 3.1–9.6 days], *p* < 0.003) [40••]. A cross-sectional study performed in the USA showed a reduction in median hospital length of stay of 7 h in the early COVID-19 period (February 23, 2020) and 6 h in the later COVID-19 period (March 29, 2020, to May 16, 2020) [19]. Early discharge of ACS patients increased bed capacity, preserved resources in an already overburdened healthcare system, and minimized the risk of exposure of ACS patients to COVID-19 in-hospital. Therefore, local protocols have been developed to aim for “very early discharge” in STEMI patients, defined as discharge 24 h after primary PCI in low-risk patients. For example, the Ottawa Heart Institute in Canada introduced the Very Early Hospital Discharge (VEHD) protocol [41]. Earlier research showed that early discharge (24 h to 3 days) after primary PCI was safe, as shown in a meta-analysis including more than 1500 STEMI patients [42]. Reports using observational data have suggested that very early discharge after 24 h is indeed safe in low-risk STEMI patients after successfully performed primary PCI [43–46].

## Impact on (Long-Term) Clinical Outcomes

Clinical outcomes for ACS patients were significantly worse during the first wave in 2020 compared with the same period in 2019. A meta-analysis showed that in-hospital mortality of STEMI patients was increased by 33%, as estimated from 34 studies [21]. For NSTEMI patients, the increase in estimated in-hospital mortality was 34% using data from 8 studies [21].

Increased mortality rates can be explained by several reasons. In STEMI patients, there was a considerable treatment delay mostly caused by patient delay, as outlined above. Indeed, in STEMI patients, this led to an increased rate of patients presenting in cardiogenic shock of 33% and an increased rate of patients with mechanical complications of 80% [21]. Some studies even reported odds ratios of 2.3 up to as high as 3.6 for patients presenting with mechanical complications during the 2020 COVID pandemic compared with the same period in 2019 [47–50]. In NSTEMI patients, worse outcomes may be explained by a higher rate of conservative therapy without invasive angiography, lower reperfusion therapy rates, and longer time to first medical contact [37].

Finally, COVID-19 infection in patients with ACS resulted in a poorer prognosis compared with ACS patients without concomitant COVID-19 infections [34, 51] and may also be an important explanation for worse outcomes during the COVID-19 pandemic.

## Conclusions

**Fig. 1:**
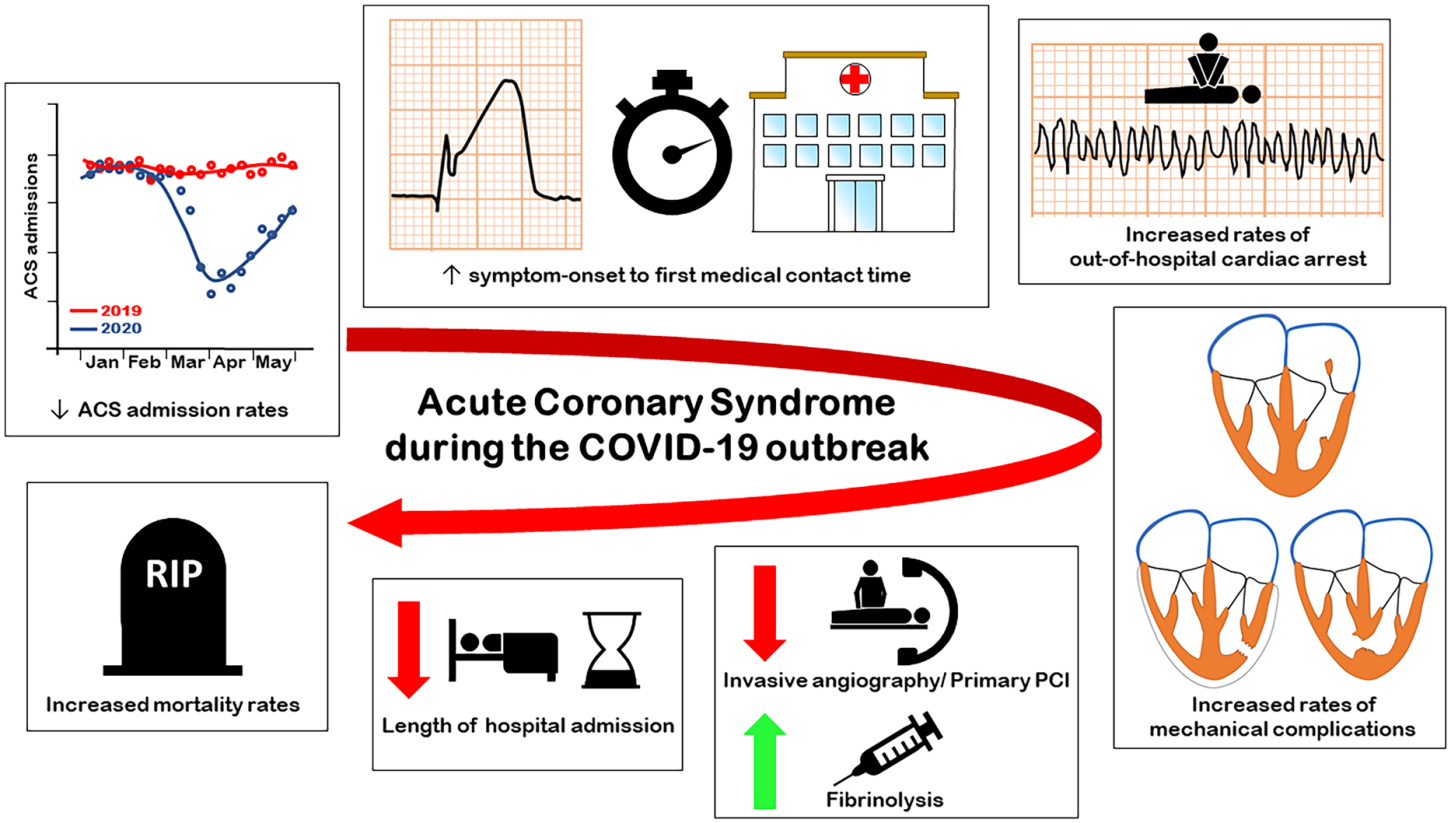
Graphical overview of the impact of the COVID-19 pandemic on the management of acute coronary syndrome. ACS, acute coronary syndrome; PCI, percutaneous coronary intervention; COVID-19, coronavirus disease 2019.

The COVID-19 pandemic has had a major impact on the management of ACS care, as summarized in Fig. 1. While an increased rate of ACS during such a pandemic is plausible (due to an increase in ACS incidence triggered by COVID-19 infections), a decreased incidence of ACS and admissions rates for ACS were observed. Although some potential ACS triggers were indeed reduced, such as less vigorous exercise due to closure of sport facilities, less air pollution, and less respiratory virus infections such as the flu, a change in patient behavior with avoidance of seeking medical help when having ACS symptoms is very likely, leading to increased symptom onset to first medical contact times and an increased rate of out-of-hospital cardiac arrests and mechanical complications of myocardial infarction. Less patients with NSTEMI underwent invasive coronary angiography, while in STEMI patients, more patients received a fibrinolysis-first management (instead of primary PCI), although a large variation was observed with some centers having a relative increase in early invasive management. Patients with ACS and concomitant COVID-19 infection have worse outcomes compared to ACS patients without concomitant COVID-19 infection. All of the above led to worse clinical outcomes in patients presenting with ACS during the COVID pandemic. Very early discharge (24 h after primary PCI) in low-risk STEMI patients on the other hand had a very good prognosis and resulted in significantly shorter hospital duration of stay. Such initiatives, and strategies to lower the reluctance of patients with ACS symptoms to seek medical help, are vital to improve prognosis in ACS patients in future pandemics.
